# Adsorption Performance of Amino Functionalized Magnetic Molecular Sieve Adsorbent for Effective Removal of Lead Ion from Aqueous Solution

**DOI:** 10.3390/nano11092353

**Published:** 2021-09-11

**Authors:** Chuanen Guo, Yingying Wang, Fangzheng Wang, Yaoguang Wang

**Affiliations:** 1 Shandong University of Political Science and Law, Jinan 250014, China; 000807@sdupsl.edu.cn; 2 Shandong Provincial Key Laboratory of Molecular Engineering, School of Chemistry and Chemical Engineering, Qilu University of Technology (Shandong Academy of Sciences), Jinan 250353, China; yingyingw1203@163.com (Y.W.); wangfzhyx@163.com (F.W.)

**Keywords:** adsorption, amino functionalized CoFe_2_O_4_, SBA–15, magnetic separation, lead ion

## Abstract

Lead ion (Pb^2+^) has high toxicity and brings great harm to human body. It is very important to find an effective method to address lead ion pollution. In this work, amino functionalized CoFe_2_O_4_/SBA–15 nanocomposite (NH_2_–CoFe_2_O_4_/SBA–15) was prepared for the effective removal of Pb^2+^ from aqueous solution. The prepared NH_2_–CoFe_2_O_4_/SBA–15 adsorbent was manifested by using scanning electron microscope (SEM), energy dispersive spectroscopy (EDS), Fourier transform infrared spectrum (FTIR), X-ray powder diffraction (XRD), and Brunauer-Emmett-Teller (BET) analysis. In the meantime, the adsorption conditions, including pH, adsorbent dosage, and adsorption time, were studied. The investigation of adsorption kinetics revealed that the adsorption results conform to the pseudo-first-order kinetic model. The adsorption isotherms research displayed that the adsorption was consistent with the Freundlich model, demonstrating that the adsorption for Pb^2+^ with the prepared adsorbent was a multimolecular layer adsorption process. In addition, the thermodynamic investigations (Δ*G* < 0, Δ*H* > 0, Δ*S* > 0) demonstrated that the adsorption for Pb^2+^ with the prepared adsorbent was endothermic and spontaneous. Moreover, the prepared adsorbent showed superior anti-interference performance and reusability, implying the potential application of the adsorbent in actual water treatment. Furthermore, this research may provide a reference and basis for the study of other heavy metal ions.

## 1. Introduction

In the process of industrial production, the substandard discharge of wastewater releases a variety of heavy metal ions into the natural environment. A great quantity of heavy metal ions become one of the main environmental pollutants [1,2]. These dissolved heavy metal ions in water can cause adverse effects on aquatic ecosystems. Long−term exposure to water which has been contaminated by heavy metals can cause serious harm to human health [3,4,5,6]. Lead ion (Pb^2+^) is a common pollutant in industrial wastewater, which can cause human dysfunction or serious lesions if ingested in large quantities or for a long time [7,8]. For example, Pb^2+^ can cause liver and kidney damage, affect the production of human hemoglobin, damage the human nervous system, cause mental retardation in infants, cause infertility and fetal malformation, etc. [9]. The general techniques for the removal of heavy metal ions in water include precipitation [10], ion exchange [11], reverse osmosis [12], nanofiltration [13], adsorption [14], etc. Among them, the adsorption method has gained extensive attention due to its simple, low cost and can overcome some potential environmental problems [15,16,17,18].

Since adsorbents with large specific surface area have a strong adsorption capacity, many researches with regard to adsorbents have focused on materials with a large specific surface area [19,20,21,22]. A molecular sieve is a kind of material with large surface area. Moreover, it also possesses the advantages of stable physical and chemical properties, uniform pore size distribution, and easy to achieve chemical modification, etc. [23,24,25,26]. Therefore, a molecular sieve is a kind of adsorbing material with great potential. SBA–15 is a typical molecular sieve with a uniform mesoporous structure [27,28]. The mesoporous pore provides enough space for chemical modification of grafted functional groups, while sufficient wall thickness makes it exhibit superior mechanical stability and hydrothermal stability than similar materials [29,30]. These above properties provide reliable guarantee for designing adsorbents. The development of mesoporous silica adsorbents functionalized with appropriate functional groups is a prospective research area for eliminating heavy metal ions away from water resources [31,32].

**Figure 1:**
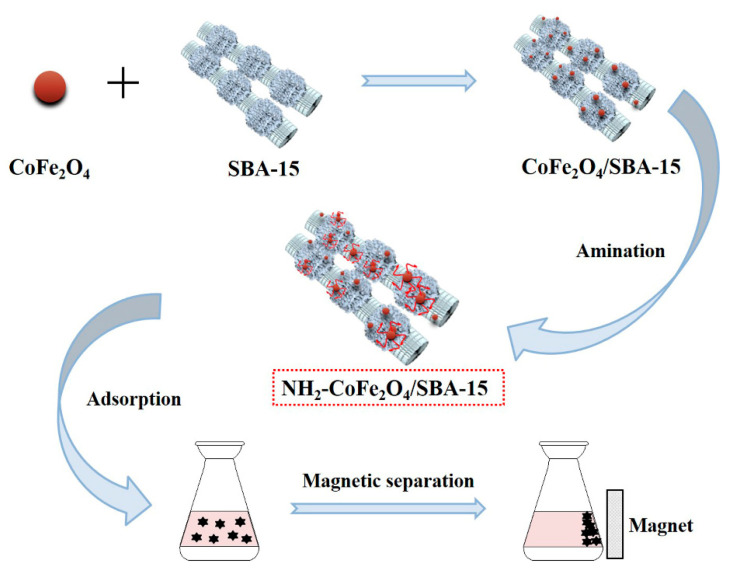
The schematic diagram for the application of NH_2_–CoFe_2_O_4_/SBA–15 toward Pb^2+^ adsorption in the presence of external magnetic field.

For the purpose of improving solid-liquid separation efficiency toward the adsorbent [33,34], we prepared the magnetic molecular sieve adsorbent by compositing SBA–15 and magnetic CoFe_2_O_4_, which could be separated well from water through an external magnetic field. To improve the adsorption capacity of the adsorbent, the surface of the prepared CoFe_2_O_4_/SBA–15 nanocomposite was aminated to enhance the complexation ability toward heavy metal ions. Herein, the adsorption properties of amino functionalized CoFe_2_O_4_/SBA–15 nanocomposite (NH_2_–CoFe_2_O_4_/SBA–15) for Pb^2+^ were investigated by using single factor experiment to optimize the adsorption process (Figure 1). Meanwhile, the adsorption kinetics, adsorption isotherm model and thermodynamic studies were investigated. Moreover, the prepared NH_2_–CoFe_2_O_4_/SBA–15 adsorbent exhibited excellent selectivity and reusability for adsorbing Pb^2+^, indicating the potential application of the adsorbent in the field of environmental protection.

## 2. Experimental Section

### 2.1. Apparatus and Reagents

SBA–15 (CAS Number: 12173-28-3, pore diameter: 6–11 nm) was obtained from XFNANO Materials Tech Co., Ltd. (Nanjing, China). Fe(NO_3_)_3_ was purchased from Sinopharm Chemical Reagent Beijing Co., Ltd. (Beijing, China). Co(NO_3_)_2_·6H_2_O was obtained from Macklin Biochemical Co., Ltd. (Shanghai, China). 3-aminopropyltriethoxysilane was purchased from Aladdin Bio-Chem Technology Co., Ltd. (Shanghai, China). Ultrapure water was applied in all experiment for the preparation of solution. All other reagents were employed with analytical reagent grade and used without further purification.

Scanning electron microscope (SEM) pictures were conducted with field-emission SEM (Gemini 300, Zeiss, Jena, Germany). Fourier transform infrared spectrum (FTIR) spectra was obtained by using KBr pellet method ranging from 4000 cm^−1^ to 400 cm^−1^ with Perkin−Elmer Spectrum One FTIR spectrometer (Perkin−Elmer, Waltham, MA, USA). X-ray powder diffraction (XRD) pattern was obtained by using a D8 FOCUS X−ray diffraction spectrometer (Bruker, Karlsruhe, Germany), together with a Cu Kα target at a scanning rate of 0.03° 2θ s^−1^ in the range of 10–80°. Brunauer-Emmett-Teller (BET) analysis was conducted by using Micromeritics ASAP 2020 surface area and porosity analyzer (Quantachrome, Boynton Beach, FL, USA). The atomic absorption spectrophotometry was proceeded by using an atomic absorption spectrophotometer (Perkin-Elmer, Waltham, MA, USA).

### 2.2. Synthesis of Magnetic CoFe_2_O_4_/SBA–15

CoFe_2_O_4_/SBA–15 was synthesized by one step immersion method as follows: Firstly, 0.116 g of Co(NO_3_)_2_·6H_2_O and 0.323 g of Fe(NO_3_)_3_ were mixed in three-neck flask with 20 mL of ultrapure water. Then 0.2 g of SBA–15 was added into the above solution with stir for mixing well. Afterwards, the flask was moved to the oil bath under 50 °C with continuous agitation. After the water in flask been evaporated to dryness, the obtained solid was moved to crucible and calcined for 5 h at 800 °C. Eventually, the magnetic CoFe_2_O_4_/SBA–15 could be obtained.

### 2.3. Synthesis of Amino Functionalized CoFe_2_O_4_/SBA–15

0.5 g of CoFe_2_O_4_/SBA–15 was dispersed into 50 mL of absolute ethanol. Then 1 mL of 3-aminopropyltriethoxysilane (APTES) was injected into the solution, followed with mechanically stirring and maintaining in oil bath for 24 h. The obtained solid was separated under magnetic field and rinsed with ethanol several times. After being disposed in drying oven at 60 °C, the product was grinded and the NH_2_–CoFe_2_O_4_/SBA–15 could be obtained.

### 2.4. Single Factor Static Adsorption Experiment

This research was conducted by using single factor static adsorption method for investigating the adsorption of Pb^2+^ onto NH_2_–CoFe_2_O_4_/SBA–15. The adsorption experiment was proceeded in 100 mL of conical flask at ambient temperature by placing the conical flask in a thermostatic water bath oscillator, along with Pb^2+^ solution (30 mg·L^−1^, 25 mL). 0.1 mol·L^−1^ of NaNO_3_ was injected into the Pb^2+^ solution as constant back−ground electrolyte. The effect of adsorbent dosage for adsorbing Pb^2+^ was operated by using different amounts of adsorbent contacting with Pb^2+^ for 3 h. The pH was adjusted by using HCl and NaOH for studying the influence of pH toward the adsorption of Pb^2+^. The adsorbent could be separated rapidly from solution by a magnet. The residual Pb^2+^ concentration in equilibrium was monitored by using atomic absorption spectrophotometry. Furthermore, the interfering ion experiment and the reusability of the prepared adsorbent were also investigated.

The removal efficiency as well as the adsorbing capacity were calculated based on the changes of Pb^2+^ concentration before and after the adsorption on the basis of the following calculation method:

(1) $$R=\frac{c_{0}-c_{e}}{c_{0}}\times 100\%$$

(2) $$q_{e}=\frac{(c_{0}-c_{e})\times V}{m}$$

where *c*_o_ (mg·L^−1^) and *c*_e_ (mg·L^−1^) represent the initial and equalized contents of Pb^2+^, respectively. *R* (%) is the removal efficiency and *q*_e_ (mg·g^−1^) is the adsorption capacity when the adsorption becomes equilibrium. The volume of Pb^2+^ solution is expressed as *V* (L) and the mass of adsorbent is described as *m* (g).

## 3. Results and Discussion

### 3.1. Characterization of NH_2_–CoFe_2_O_4_/SBA–15

**Figure 2:**
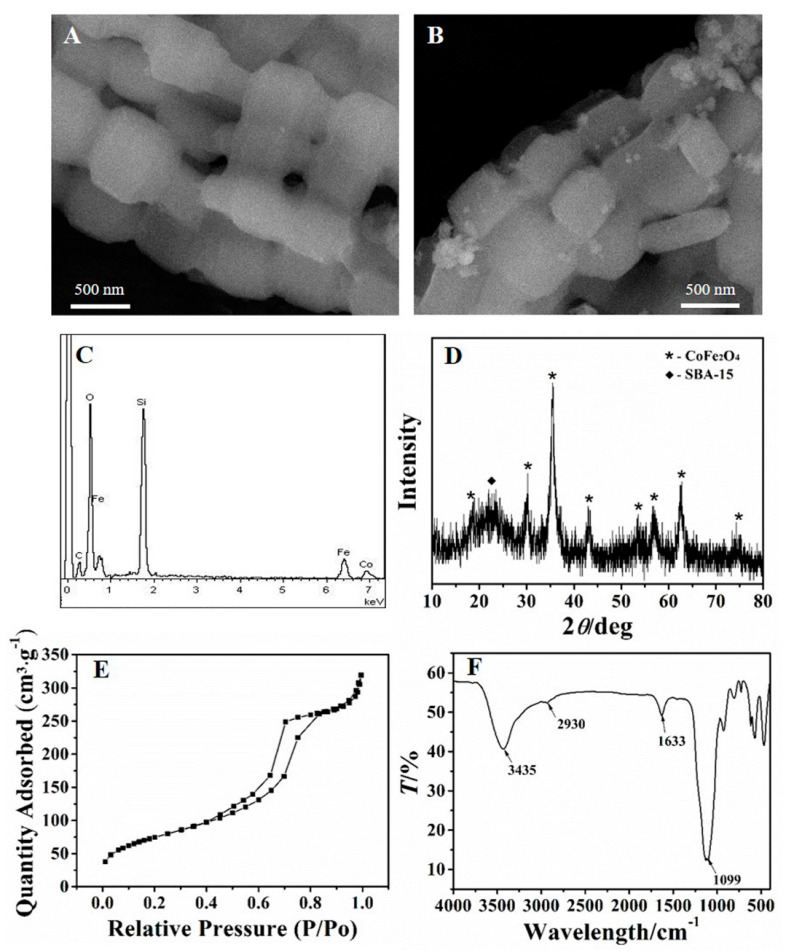
SEM images of SBA–15 (**A**) and CoFe_2_O_4_/SBA–15 (**B**); EDS spectrum of CoFe_2_O_4_/SBA–15 (**C**); XRD pattern (**D**), BET analysis (**E**), and FTIR spectrum (**F**) of NH_2_–CoFe_2_O_4_/SBA–15.

Figure 2A,B show the morphology of SBA–15 and CoFe_2_O_4_/SBA–15. As can be seen from Figure 2A, the SBA–15 owned chaplet-like shape. Observed from Figure 2B, the surface of SBA–15 appeared new nanoparticles, which was due to the formation of CoFe_2_O_4_. Furthermore, energy dispersive spectroscopy (EDS) was applied to analysis the component of the adsorbent. As Figure 2C shows, the adsorbent contained not only the elements of Si and O, but also contained Fe and Co, demonstrating the existence of constituent elements for CoFe_2_O_4_/SBA–15.

The XRD pattern was further used to analysis the NH_2_–CoFe_2_O_4_/SBA–15. As shown in Figure 2D, the dispersion peak nearly 2θ = 23° was attributed to the amorphous SBA–15. The other diffraction peaks in the figure were consistent with the spectrum of CoFe_2_O_4_ [35]. According to the above results, it could conclude that the CoFe_2_O_4_ was loaded on SBA–15 successfully.

Figure 2E shows the BET analysis of NH_2_–CoFe_2_O_4_/SBA–15. Since the existence of the adsorption and desorption hysteresis loop, it is obvious that the NH_2_–CoFe_2_O_4_/SBA–15 owns uniform and orderly mesoporous channels, which is beneficial for adsorbing Pb^2+^. The specific surface area which was measured for the prepared NH_2_–CoFe_2_O_4_/SBA–15 was about 273.8 m^2^·g^−1^, which was lower than pure SBA–15 (about 550 m^2^·g^−1^). This might be due to the fact that the introduction of CoFe_2_O_4_ blocked the pores of SBA–15, and thus the specific surface area of NH_2_–CoFe_2_O_4_/SBA–15 decreased.

Figure 2F displays the FTIR spectrum of NH_2_–CoFe_2_O_4_/SBA–15. As shown in the spectrum, the bond of N–H stretching vibration can be testified by the peak at 3435 cm^−1^, while the peak at the position of 1633 cm^−1^ belonged to the deformation vibration absorption peak of N–H. The peaks at 2930 cm^−1^ were the absorption peak of C–H. The peak at 1099 cm^−1^ was the Si–O absorption peak of SBA–15. The results further illustrated the successful synthesis of NH_2_–CoFe_2_O_4_/SBA–15.

### 3.2. Influence of pH toward Removal Efficiency

The pH of the employed solution is considered to be a critical ingredient to impact the adsorption process [36]. The impact is mainly reflected in the following aspects. Firstly, the solution pH could affect the charge type and charge number on the adsorbent, which determines whether the adsorption process can either occur or not occur. Secondly, the charge number on the adsorbent surface determines the molar ratio of adsorbent and adsorbate when it reaches saturated adsorption. Thirdly, the pH could influence the existing form of pollutant, further affect the state of adsorbent or pollutant, which determines the mechanism of the adsorption process.

**Figure 3:**
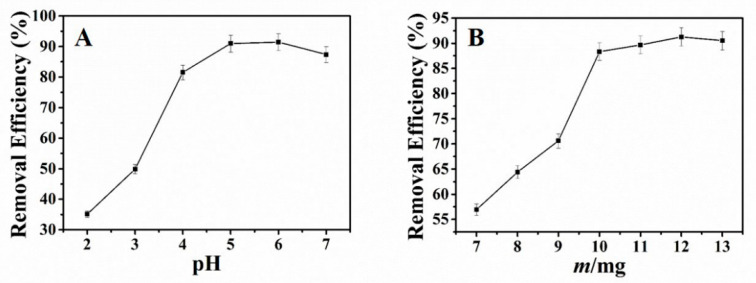
Effect of pH (**A**) and adsorbent dosage (**B**) on adsorption of Pb^2+^ (initial content of Pb^2+^: 25 mL 30 mg·L^−1^; time: 3 h; temperature: ambient temperature).

In this study, we mainly investigated the effect of pH toward the adsorption of Pb^2+^ with NH_2_–CoFe_2_O_4_/SBA–15. The experiment was conducted as follows: 25 mL 30 mg·L^−1^ of Pb^2+^ solution was added into the 100 mL conical flask. HNO_3_ and NaOH were applied to adjust the pH. Then 10 mg of adsorbent was added and vibrated for 3 h at room temperature. The residual Pb^2+^ was measured and the removal efficiency could be obtained. Observed from Figure 3A, when the pH of the solution was under 4.0, the efficiency for the removal of Pb^2+^ was very low. With the increase of the pH, the removal efficiency increased. And the maximum removal efficiency was from pH 5.0 to pH 6.0. This result could be interpreted as follows: When at low pH, the H^+^ and Pb^2+^ in solution were competitive with each other. And the H^+^ could protonate the –NH_2_ on the surface of adsorbent. The surface of the adsorbent is positively charged, which could repel with Pb^2+^ because of the static electricity. Furthermore, the H^+^ hindered the coordination between Pb^2+^ and adsorbent, decreasing the adsorption capacity of the adsorbent. However, when the pH increased, the –NH_2_ on the surface of adsorbent deprotonated. Then the –NH_2_ as electron-rich group would attract a positively charged Pb^2+^ and complex with it. Thus, the adsorption capacity increased. According to the experimental results, the optimal pH 5.0 was chosen for future experiment.

### 3.3. Effect of Adsorbent Dosage on the Removal Efficiency

Selected amount of NH_2_–CoFe_2_O_4_/SBA–15 were added into 100 mL round-bottom flask with 25 mL 30 mg·L^−1^ of Pb^2+^ (pH 5.0) and agitated for 3 h at ambient temperature. The influence of adsorbent dosage toward the removal of Pb^2+^ displays in Figure 3B. When the adsorbent dosage aggrandized, the removal efficiency for Pb^2+^ fortified. As the adsorbent dosage was 10 mg, the removal efficiency became very high and changed little with the increase of the adsorbent dosage. Thus, the adsorbent dosage of 10 mg was selected in the subsequent experiment.

### 3.4. Influence of Adsorption Time and Adsorption Kinetics

**Figure 4:**
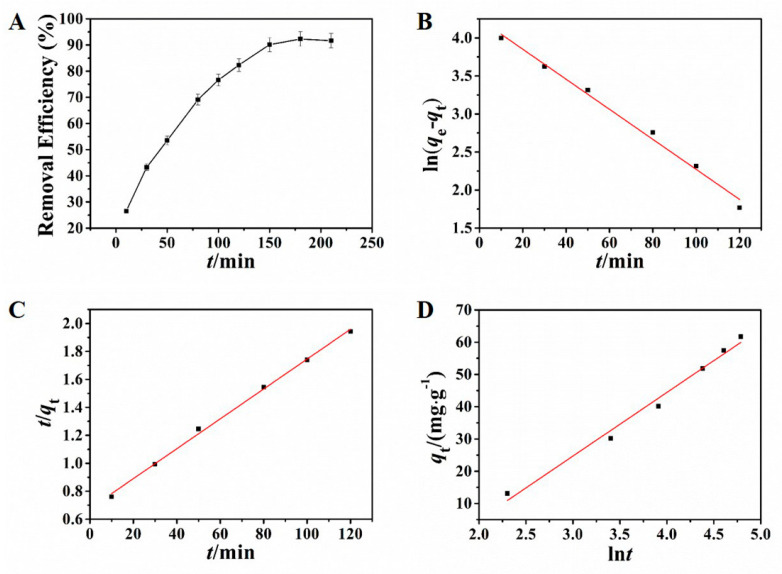
Influence of adsorption time toward the removal efficiency (**A**); pseudo-first-order kinetic model (**B**), pseudo-second-order kinetic model (**C**), and Elovich kinetic model (**D**) for adsorption of Pb^2+^.

Adsorption kinetics resulting from the adsorption process are crucial in the process of studying adsorption behavior, which reflects the basic information of adsorption rate and reaction routes [37,38]. The effect of adsorption time was investigated in this study. In the condition of the selected pH and adsorbent dosage in the preliminary experiment, different adsorption times were applied to investigate the adsorption of Pb^2+^ by using NH_2_–CoFe_2_O_4_/SBA–15. As shown in Figure 4A, the adsorption process reached equilibrium at 150 min.

Adsorption kinetics has been an important aspect for study the adsorption mechanism. Herein, the pseudo-first-order kinetic model, pseudo-second-order kinetic model, and Elovich kinetic model were employed to explore the dynamics features for the adsorption behavior. Each model could be presented below:

Pseudo-first-order kinetic model:

(3) $$\ln(q_{e}-q_{t})=\ln q_{e}-k_{1}t$$

Pseudo-second-order kinetic model:

(4) $$\frac{t}{q_{t}}=\frac{1}{k_{2}q_{e}^{2}}+\frac{t}{q_{e}}$$

Elovich kinetic model:

(5) $$q_{t}=\frac{1}{\beta }\ln\left(\alpha \cdot \beta \right)+\frac{1}{\beta }\ln\left(t/\min\right)$$

here *q*_t_ (mg·g^−1^) represents the adsorption capacities at time *t* (min). *q*_e_ (mg·g^−1^) represents the adsorption capacities at equilibrium adsorption. The pseudo-first-order rate constant and pseudo-second-order rate constant are denoted as *k*_1_ (min^−1^) and *k*_2_ (g·mg^−1^·min^−1^) separately. The initial adsorption rate in Elovich model is defined as *α* (mg·g^−1^·min^−1^), while the desorption constant is expressed as *β* (g·mg^−1^).

The kinetic results are displayed in Figure 4B–D and the corresponding calculative results were presented in Table 1. Observed from the matching results, either the pseudo-first-order kinetic model or the pseudo-second-order kinetic model had high linear coefficient. However, the test result of the equilibrium adsorbing amount was 67.61 mg·g^−1^, close to the result from the pseudo-first-order kinetic model (69.78 mg·g^−1^). The relative error was only 3.2%, indicating that the adsorption kinetic process can be fitted splendidly by pseudo-first-order kinetic model, indicating that the adsorption behavior was dominated by diffusion step.

### 3.5. Adsorption Isotherms Study

Herein, four kinds of adsorption isotherm models were applied to investigate the surface behavior of the prepared adsorbent in the solution:

Henry model:

(6) $$q_{e}=kc_{e}$$

Langmuir model:

(7) $$\frac{1}{q_{e}}=\frac{1}{bq_{m}c_{e}}+\frac{1}{q_{m}}$$

Freundlich model:

(8) $$\ln q_{e}=\ln K_{F}+\frac{1}{n}\ln c_{e}$$

Temkin model:

(9) $$q_{e}=\frac{RT}{b_{T}}\ln c_{e}+\frac{RT}{b_{T}}\ln A_{T}$$

where *q*_m_ (mg·g^−1^) represents the maximum adsorption capacity. *b* (L·mg^−1^) represents Langmuir constant about the binding capacity of the binding site. *k* (L·g^−1^) and *K*_F_ (L·g^−1^) represent the corresponding constants for adsorption capacity and strength. $\frac{RT}{b_{T}}$ is relevant toward the adsorption heat. *A*_T_ (L·g^−1^) represents the constant in equilibrium related with the supreme binding energy. *n* (dimensionless) is an empirical parameter related to the intensity of adsorption, which varies with the heterogeneity of the adsorbent.

**Figure 5:**
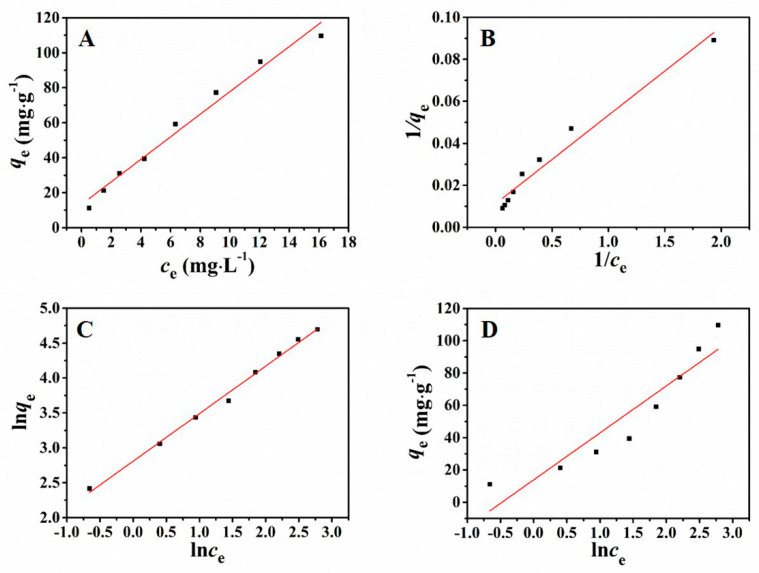
Henry (**A**), Langmuir (**B**), Freundlich (**C**), and Temkin (**D**) adsorption isotherms of Pb^2+^.

Figure 5 revealed the results of adsorption isotherms which were fitted by the above four models. The parameters which were calculated according to the four models are presented in Table 2. Observed from Table 2, the adsorption behavior could be well fitted by using the Freundlich model (*R*^2^= 0.9942) which is higher than the correlation coefficient of other models. The simulated results revealed that the adsorption behavior was consistent with the Freundlich model, manifesting that the adsorption for Pb^2+^ was a multi-molecular layer adsorption process.

The Freundlich isotherm model demonstrates that the ratio of solute adsorbed on solid surface to the solute concentration has a certain relationship toward the solution concentration. This model considers multiple types of adsorption sites on the solid and represents the adsorption data at low concentrations and intermediate concentrations on heterogeneous surfaces felicitously. And the symbol of *n* reveals that the adsorption process occurs on the heterogeneous surfaces and is a reversible course.

### 3.6. Thermodynamic Parameters Study

The influence of temperatures on Pb^2+^ adsorption was investigated under the temperatures of 298 K, 308 K, and 318 K. The thermodynamic variables were obtained by using the calculation method below:

(10) $$\Delta G=-RT\ln K_{d}$$

(11) $$\ln K_{d}=\frac{\Delta S}{R}-\frac{\Delta H}{RT}$$

Herein, *R* (8.314 J·mol^−1^·K^−1^) represents the gas constant. *T* represents the (Kelvin) temperature (*K*). *K*_d_ represents the adsorption equilibrium constant. The Gibbs free energy change is defined as Δ*G* (kJ·mol^−1^). The entropy change is expressed as Δ*S* (J·mol^−1^·K^−1^). Furthermore, Δ*H* (kJ·mol^−1^) demonstrates the enthalpy change in a given process. The obtained results of the thermodynamic parameters were displayed in Table 3. From the table, it can be seen that the Δ*G* was less than 0, indicating the spontaneous adsorption process for the removal of Pb^2+^. While the Δ*H* with positive value manifested the endothermic process of the adsorption for Pb^2+^ by using the prepared adsorbent. Meanwhile, the positive value of Δ*S* indicated the increase in degree of chaos at the interface of solid and solution as well as the good affinity of Pb^2+^ with NH_2_–CoFe_2_O_4_/SBA–15.

### 3.7. Evaluation of Adsorption Performance

**Figure 6:**
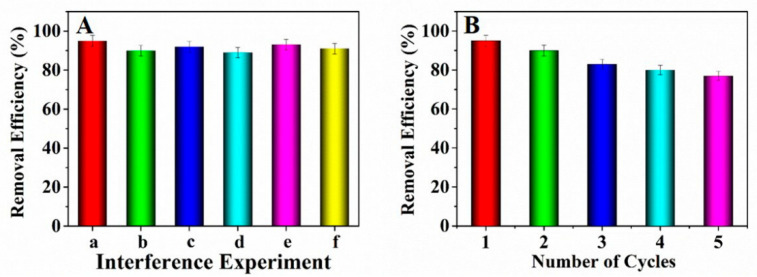
(**A**) Influence of interfering ions for Pb^2+^ adsorption (a: blank; b: Na^+^; c: K^+^; d: Mg^2+^; e: Ca^2+^; f: mixture of the above ions); (**B**) Reusability about the prepared adsorbent.

The existing interfering ions including Na^+^, K^+^, Mg^2+^ and Ca^2+^ might influence the adsorption of Pb^2+^. Hence the adsorption interference experiments were conducted by adding NaNO_3_, KNO_3_, Mg(NO_3_)_2_ and Ca(NO_3_)_2_ into the solutions containing lead ions, respectively. Observed from Figure 6A, the concomitant interfering ions almost had no influence on the Pb^2+^ adsorption. Therefore, it could be concluded that the prepared adsorbent can adsorb Pb^2+^ effectively.

The reusability is an important index to investigate the property of the adsorbent [39]. Herein, to investigate the reusability of the prepared adsorbent, an adsorption-desorption experiment was conducted by adding the adsorbent which has adsorbed Pb^2+^ into 15 mL of NaOH solution (0.1 mol·L^−1^) and maintained for 3 h. Afterwards, the adsorbent was washed with ultrapure water to remove the excess alkali solution for three times. Observed from Figure 6B, five times of adsorption-desorption cycles were studied. The consequences showed that the adsorption efficiency decreased to some extent. After five cycles, the removal efficiency kept at above 77%, indicating that the prepared adsorbent NH_2_–CoFe_2_O_4_/SBA–15 possessed good performance as recyclable adsorbent in dealing with the wastewater containing Pb^2+^.

## 4. Conclusions

In conclusion, a novel NH_2_–CoFe_2_O_4_/SBA–15 adsorbent was prepared and applied in the adsorption of Pb^2+^. The adsorption results revealed that the adsorption behavior intensively relied on the pH of the solution with an adsorbent dosage of 10 mg and pH 5.0. The results of adsorption kinetics research can be well described by the pseudo-first-order kinetic model, revealing that the adsorption process was controlled by diffusion step. Meanwhile, the adsorption isotherms research indicated that the adsorption behavior could be expressed better through the Freundlich model. The thermodynamic studies (Δ*G* < 0, Δ*H* > 0, Δ*S* > 0) revealed the endothermic and spontaneous character of the adsorption behavior. The excellent selectivity and reusability of the prepared adsorbent made it a promising adsorbing material in the treatment of actural wastewater containing lead ions. However, the prepared adsorbent could only adsorb the single Pb^2+^. If the adsorbent could interact with other contaminants simultaneously and effectively, it will greatly expand its application in the field of environmental protection.

## Figures and Tables

**Table 1:** Kinetic model parameters for the adsorption of Pb^2+^.

Models	Parameters	
Pseudo-first-order kinetic model	*q*_e_ (mg·g^−1^)	69.78
	*k*_1_ (min^−1^)	0.01974
	*R* ^2^	0.9898
Pseudo-second-order kinetic model	*q*_e_ (mg·g^−1^)	94.34
	*k*_2_ (g·mg^−1^·min^−1^)	0.0001660
	*R* ^2^	0.9974
Elovich kinetic model	*α* (mg·g^−1^·min^−1^)	3.436
	*β* (g·mg^−1^)	0.05071
	*R* ^2^	0.9842

**Table 2:** Parameters of adsorption isotherm for adsorbing Pb^2+^ with NH_2_–CoFe_2_O_4_/SBA–15.

Models	Parameters	
Henry	*k*	6.447
	*R* ^2^	0.9782
Langmuir	*q*_m_ (mg·g^−1^)	89.13
	*b* (L·mg^−1^)	0.2664
	*R* ^2^	0.9641
Freundlich	*K*_F_	16.71
	*n*	1.470
	*R* ^2^	0.9942
Temkin	*b*_T_	88.0
	*A*_T_	1.613
	*R* ^2^	0.8659

**Table 3:** Thermodynamic parameters for adsorption of Pb^2+^ with NH_2_–CoFe_2_O_4_/SBA–15.

*T* (K)	Δ*G* (kJ·mol^−1^)	Δ*S* (J·mol^−1^·K^−1^)	Δ*H* (kJ·mol^−1^)
298	−5.45	121.5	30.76
308	−6.66		
318	−7.88		

## Data Availability

The data presented in this study are available on request from the corresponding author.
